# A123 THE EFFECTS OF INFLAMMATORY BOWEL DISEASE ON THE MANAGEMENT AND OUTCOMES OF COMPLEX POLYPS IN PATIENTS UNDERGOING SURVEILLANCE COLONOSCOPY: A RETROSPECTIVE COHORT STUDY

**DOI:** 10.1093/jcag/gwad061.123

**Published:** 2024-02-14

**Authors:** R AlRamdan, A Almudaires, S Son, M Sey, J Gregor, B Yan

**Affiliations:** University of Western Ontario Schulich School of Medicine & Dentistry, Western University Schulich School of Medicine & Dentistry, London, ON, CA, academic/medsch, London, ON, Canada; University of Western Ontario Schulich School of Medicine & Dentistry, Western University Schulich School of Medicine & Dentistry, London, ON, CA, academic/medsch, London, ON, Canada; University of Western Ontario Schulich School of Medicine & Dentistry, Western University Schulich School of Medicine & Dentistry, London, ON, CA, academic/medsch, London, ON, Canada; University of Western Ontario Schulich School of Medicine & Dentistry, Western University Schulich School of Medicine & Dentistry, London, ON, CA, academic/medsch, London, ON, Canada; University of Western Ontario Schulich School of Medicine & Dentistry, Western University Schulich School of Medicine & Dentistry, London, ON, CA, academic/medsch, London, ON, Canada; University of Western Ontario Schulich School of Medicine & Dentistry, Western University Schulich School of Medicine & Dentistry, London, ON, CA, academic/medsch, London, ON, Canada

## Abstract

**Background:**

The influence of inflammatory bowel disease (IBD) on the outcomes of complex polyps remains an area of uncertainty.

**Aims:**

To investigate the impact of IBD on the management and outcomes of complex polyps during surveillance colonoscopy.

**Methods:**

This retrospective cohort study utilized a prospectively collected database, involving patients who underwent surveillance colonoscopy for colorectal cancer or IBD surveillance who were identified to have complex polyps between February 2019 and December 2020 in Southwestern Ontario. Patient demographics, disease characteristics, medication history, polyp description and management were analyzed. The data were summarized in median and interquartile range for continuous variable and frequency and percentage for categorical variables. Characteristics between IBD and non-IBD were compared using the Wilcoxon rank sum test and Fisher’s exact test, as appropriate.

**Results:**

383 patients were included in the study, of which 27 individuals had IBD (5 Crohn's disease, 13 ulcerative colitis, and 9 indeterminate colitis). Patients with IBD were generally younger than their non-IBD counterparts (median age 61 vs 67 respectively). No significant differences were identified in terms of rates of complete polyp removal (17.70 % vs 22.22 %, pampersand:003C0.602), residual polyps after resection (76.97% vs 70.37%, pampersand:003C0.476), repeat removal attempts (84.27% vs 74.07%, pampersand:003C0.179), or surgical intervention (88.76% vs 88.89%, pampersand:003C0.99). A higher proportion of IBD patients were managed by gastroenterologists vs other endoscopists (81.48% vs. 40.17%, p ampersand:003C 0.001). No significant differences in complex polyp characteristics were observed, except in difficult locations of complex polyps were more common in the non-IBD cohort (55.34% vs 25.93%, pampersand:003C0.004).

**Conclusions:**

In this retrospective cohort study, IBD does not appear to influence the management or outcome of complex polyps. Larger studies are required given the small IBD cohort size in this study.

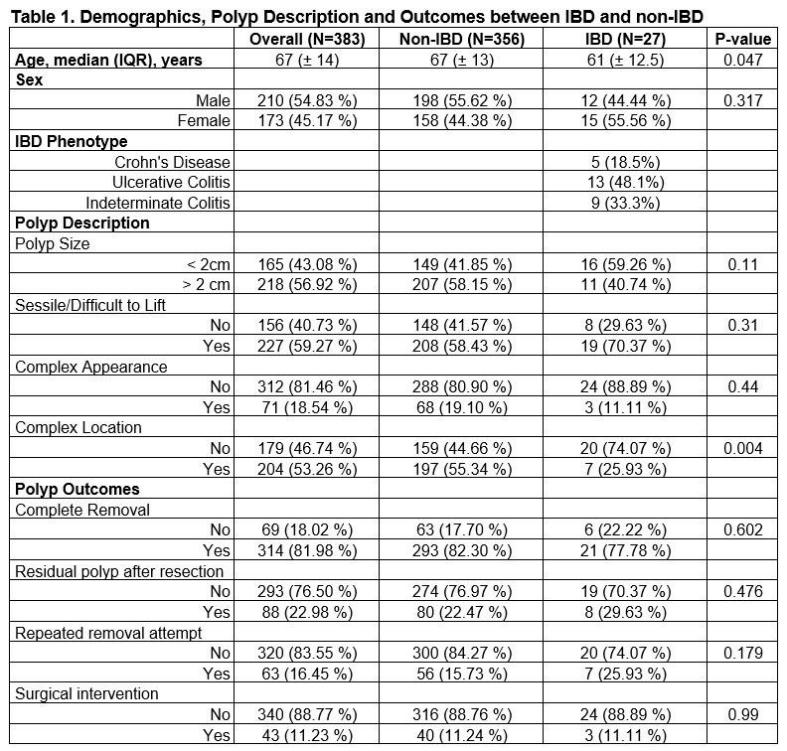

**Funding Agencies:**

None

